# π-Stacked Polymer Consisting of a Pseudo–*meta*–[2.2]Paracyclophane Skeleton

**DOI:** 10.3390/polym10101140

**Published:** 2018-10-12

**Authors:** Hazuki Maeda, Mayu Kameda, Takuji Hatakeyama, Yasuhiro Morisaki

**Affiliations:** 1 Department of Applied Chemistry for Environment, School of Science and Technology, Kwansei Gakuin University, 2-1 Gakuen, Sanda, Hyogo 669-1337, Japan; ebk97584@kwansei.ac.jp; 2 Department of Chemistry, School of Science and Technology, Kwansei Gakuin University, 2-1 Gakuen, Sanda, Hyogo 669-1337, Japan; mqu.oq@kwansei.ac.jp (M.K.); hatake@kwansei.ac.jp (T.H.)

**Keywords:** [2.2]paracyclophane, π-stacked polymer, through-space conjugation

## Abstract

A novel π-stacked polymer based on a pseudo–*meta*–linked [2.2]paracyclophane moieties was synthesized by Sonogashira-Hagihara coupling. The UV-vis absorption spectra of the synthesized polymer and model compounds revealed an extension of the conjugation length owing to the through-space conjugation. The optical properties of the π-stacked dimer with the pseudo–*meta*–linked [2.2]paracyclophane unit were compared with those of the corresponding dimers with the pseudo–*ortho*– and pseudo–*para*–linked [2.2]paracyclophane units.

## 1. Introduction

“Cyclophane” is the general term for cyclic compounds that include at least one aromatic ring (arylene unit) in the cyclic skeleton. [2.2]Paracyclophane is a representative cyclophane comprising of two *p*-phenylenes and two ethylene chains [1,2,3,4]. The two phenylenes are stacked in proximity; the distance between phenylene rings is approximately 3 Å. Although [2.2]paracyclophanes are well-known and have been actively studied in the field of synthetic organic chemistry, they have not been sufficiently applied in the fields of polymer and materials chemistry. 

**Figure 1:**
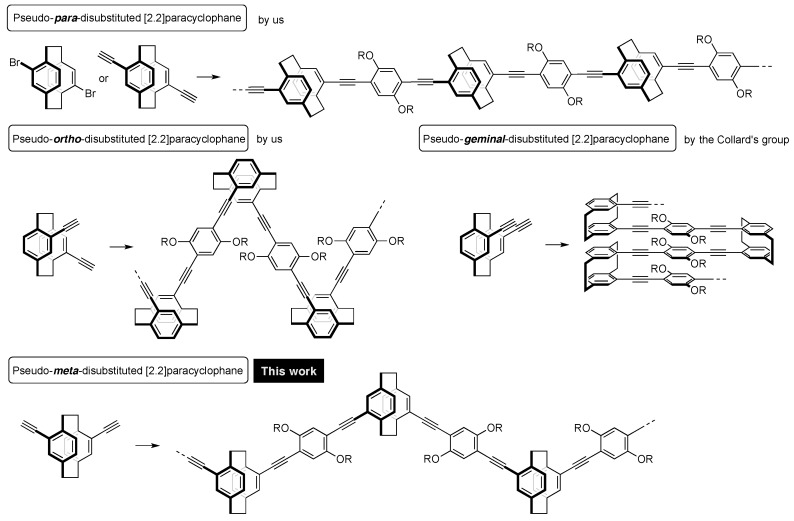
Poly(*p*-arylene-ethynylene) (PAE) type of [2.2]paracyclophane-containing through-space conjugated polymers containing various disubstituted [2.2]paracyclophane isomers.

Our interests have focused on the unique structures of [2.2]paracyclophane, repeatedly incorporating their molecular skeletons into π-conjugated polymer backbones through the use of disubstituted [2.2]paracyclophane compounds as comonomers [5,6,7,8,9,10]. For example, as shown in Figure 1, we synthesized a new type of conjugated polymer based on the pseudo–*para*–disubstituted [2.2]paracyclophane skeleton from pseudo–*para*–dibromo or diethynyl [2.2]paracyclophane [5,6,10]. The [2.2]paracyclophane unit provided a void space in the conjugated polymer main chain, leading to a partly π-stacked structure. The UV-vis absorption spectra of the corresponding oligomers as well as polymers with various molecular weights were red-shifted as the number of stacked π-electron systems increased [10]. Thus, we named this class of π-stacked polymers “through-space conjugated polymers”. 

Pseudo–*ortho*–diethynyl[2.2]paracyclophane was also used as a comonomer to prepare the corresponding zigzag-shaped through-space conjugated polymer [7]. Pseudo–*ortho*–disubstituted [2.2]paracyclophanes are planar chiral compounds. After the successful development of an optical resolution method [11], we synthesized optically active through-space conjugated polymers [12,13]. This polymer emitted intense circularly polarized luminescence by photo-excitation due to the formation of a one-handed helix in the excited state.

Collard and coworkers prepared through-space conjugated polymers consisting of the pseudo–*geminal*–disubstituted [2.2]paracyclophane backbone, in which the π-electron systems were fully π-stacked and strongly interacted with each other in the polymer [14].

With this background, our next target was very simple: That is, the synthesis of a through-space conjugated polymer incorporating pseudo–*meta*–disubstituted [2.2]paracyclophane units in the main chain. Very recently, Yu et al. reported tetraphenylethene-based conjugated polymers containing pseudo–*meta*–disubstituted [2.2]paracyclophane, which exhibited aggregation-induced emission [15]. From the viewpoint of a systematic study of poly(*p*-arylene-ethynylene) (PAE)-types of [2.2]paracyclophane-containing through-space conjugated polymers, we pursued the synthesis of the pseudo–*meta*–analogue. Herein, we report the preparation of the through-space conjugated polymer with the pseudo–*meta*–disubstituted [2.2]paracyclophane repeating units, and a comparison of its optical properties with those of the pseudo-*para*- and pseudo-*ortho*-disubstituted [2.2]paracyclophane-containing polymers.

## 2. Materials and Methods

### 2.1. Materials

[2.2]Paracyclophane (**1**), Br_2_, trimethylsilylacetylene, Pd_2_(dba)_3_ (dba = dibenzylideneacetone), (*t-*Bu)_3_P·HBF_4_, dppf (1,1’-bis(diphenylphosphino)ferrocene), CuI, and K_2_CO_3_ were commercially available compounds and used without purification. CCl_4_ and MeOH were commercially available solvents and used without purification. Super dehydrated tetrahydrofuran (THF) was purchased and used without purification. Et_3_N was used after distillation over KOH.

Compounds *p*-**2** [16,17], *m*-**2** [16,17], *m*-**3** [18], and *m*-**4** [17,18] were known compounds; however, our synthetic procedures and results are shown. Compounds **5** [19,20] and **6** [13] were prepared via procedures described in the literature. Reported data [13] of *o*-**D1** (enantiopure *R*_p_-isomer) was used in this manuscript. Synthetic route and the data of *p*-**D1** were reported [10]; however, it was prepared and the data was collected again for this study. 

### 2.2. Methods

^1^H and ^13^C NMR spectra were recorded on a JEOL JNM ECA-300 instrument at 300 MHz and a JEOL JNM ECX-500II instrument at 125 MHz, respectively. Samples were analyzed via thin layer chromatography (TLC) using silica gel 60 Merck F_254_ plates. Column chromatography was performed with Wakogel C-300 SiO_2_. Flash column chromatography and recyclable preparative high-performance liquid chromatography (HPLC) were carried out on a YMC LC Forte/R system. High-resolution mass (HRMS) spectra were obtained on a JEOL JMS-S3000 spectrometer for matrix assisted desorption/ionization (MALDI) with 7,7,8,8-tetracyanoquinodimethane (TCNQ) as a matrix. Gel permeation chromatography (GPC) was carried out on a JASCO EXTREMA GPC system with TSKgel G3000H_XL_ and G4000 H_XL_ columns using THF as an eluent after calibration with standard polystyrene samples. UV-vis spectra were recorded on a JASCO V-730 spectrophotometer, and samples were analyzed in CHCl_3_ at room temperature. Photoluminescence (PL) spectra and excitation spectra were recorded on a JASCO FP-8500 spectrofluorometer, and samples were analyzed in CHCl_3_ at room temperature. The absolute PL quantum efficiency was calculated on a JASCO FP8500 with an ILF-835 integrating sphere. The PL lifetime measurement was performed on a Hamamatsu Photonics Quantaurus-Tau fluorescence lifetime spectrometer system. Thermogravimetric analysis (TGA) was made on a Seiko EXSTAR 6000 instrument (10 °C/min) under N_2_. Differential scanning calorimetry (DSC) analysis was carried out on a Seiko DSC200 instrument at heating and cooling rate of 10 °C/min under N_2_. 

### 2.3. Synthetic Procedures

#### 2.3.1. Synthesis of *m*-**2**

[2.2]Paracyclophane (**1**) (21.7 g, 0.10 mol) was dissolved in CCl_4_ (300 mL), and Br_2_ (100 g, 0.63 mol) in CCl_4_ (30 mL) was added dropwise at 55 °C. During the reaction, a white solid was formed. After 2 h of stirring, the reaction mixture was cooled to room temperature, and the aqueous saturated solution of NaHSO_3_ was added. The white solid (*p*-**2**, 13.7 g, 38 mmol, 36%) was removed by filtration. The organic layer of the filtrate was dried by a rotary evaporator, and the residue was purified by recrystallization from hexane to obtain *m*-**2** (10.9 g, 30 mmol, 29%) as a white crystal. The ^1^H and ^13^C NMR data were matched with the reported values [16,17].

#### 2.3.2. Synthesis of *m*-**3**

A mixture of *m*-**2** (0.56 mg, 1.5 mmol), Pd_2_(dba)_3_ (0.17 g, 0.19 mmol), (*t-*Bu)_3_P·HBF_4_ (97 mg, 0.34 mmol), CuI (51 mg, 0.27 mmol), THF (20 mL) and Et_3_N (20 mL) was placed in a round-bottom flask equipped with a magnetic stirring bar. After degassing the reaction mixture several times, trimethylsilylacetylene (3.0 mL) was added to the mixture. The reaction was carried out at reflux temperature for 24 h with stirring. After the reaction mixture was cooled to room temperature, precipitates were removed by filtration, and the solvent was removed with a rotary evaporator. The residue was purified by column chromatography on SiO_2_ (CHCl_3_/hexane = 1/4 *v*/*v* as an eluent) to afford *m*-**3** (0.51 g, 1.3 mmol, 92%) as a pale yellow solid. *R*_f_ = 0.43 (CHCl_3_/hexane = 1/4 *v*/*v*). The ^1^H NMR data were matched with the reported values [18].

#### 2.3.3. Synthesis of *m*-**4**

K_2_CO_3_ (2.0 g, 14 mmol) was added to a suspension of *m*-**3** (0.81 g, 2.0 mmol) in MeOH (35 mL). After the mixture was stirred for 24 h at room temperature, H_2_O and CHCl_3_ were added to the reaction mixture. The organic layer was extracted with CHCl_3_ and washed with brine. The combined organic layer was dried over MgSO_4_. MgSO_4_ was removed by filtration, and the solvent was removed with a rotary evaporator. The residue was purified by column chromatography on SiO_2_ (CHCl_3_/hexane = 1/2 *v*/*v* as an eluent) to afford *m*-**4** (0.48 g, 1.9 mmol, 92%) as a white solid. *R*_f_ = 0.52 (CHCl_3_/hexane = 1/2 *v*/*v*). The ^1^H NMR data were matched with the reported values [17,18].

#### 2.3.4. Synthesis of *m*-**P1**

A mixture of *m*-**4** (39 mg, 0.15 mmol), **5** (11 mg, 0.15 mmol), Pd_2_(dba)_3_ (10 mg, 0.011 mmol), (*t-*Bu)_3_P·HBF_4_ (9.2 mg, 0.032 mmol), CuI (5.4 mg, 0.028 mmol), THF (1.5 mL) and Et_3_N (1.5 mL) was placed in a Schlenk tube equipped with a magnetic stirring bar. After degassing the reaction mixture several times, polymerization was carried out at reflux temperature for 48 h with stirring. After the reaction mixture was cooled to room temperature, precipitates were removed by filtration, and the solvent was removed with a rotary evaporator. The residue was purified by reprecipitation three times from CHCl_3_ and MeOH (good and poor solvent, respectively) to afford *m*-**P1** (86 mg, 0.12 mmol, 81%) as an orange solid. ^1^H NMR (CDCl_3_, 300 MHz) δ 0.86 (br), 1.24 (br), 1.56 (br). 1.96 (br), 2.99 (br), 3.12 (br), 3.24 (br), 3.66 (br), 4.11 (br), 6.53 (br), 6.66 (br), 7.07 (br), 7.17 (br) ppm; ^13^C NMR (CDCl_3_, 125 MHz) δ 14.1, 22.7, 26.2, 29.3 (m), 31.9, 33.0, 35.0, 69.5, 70.3, 88.9, 89.8, 94.5, 95.0, 114.0, 116.2, 122.7, 125.8, 128.4, 129.0, 130.8, 132.2, 132.3, 136.5, 139.3, 142.8, 153.6 ppm. ^1^H and ^13^C NMR spectra are shown in Figures S1 and S2, respectively.

#### 2.3.5. Synthesis of *m*-**D1**

A mixture of *m*-**4** (10.9 mg, 0.043 mmol), **6** (70.5 mg, 0.100 mmol), Pd_2_(dba)_3_ (5.4 mg, 0.006 mmol), dppf (8.4 mg, 0.015 mmol), CuI (3.8 mg, 0.020 mmol), THF (2.0 mL) and Et_3_N (2.0 mL) was placed in a Schlenk tube equipped with a magnetic stirring bar. After degassing the reaction mixture several times, the reaction was carried out at reflux temperature for 48 h with stirring. After the reaction mixture was cooled to room temperature, precipitates were removed by filtration, and the solvent was removed with a rotary evaporator. The residue was purified by a recyclable HPLC (CHCl_3_ as an eluent) to afford *m*-**D1** (38.5 mg, 0.027 mmol, 64%) as a yellow solid. ^1^H NMR (CDCl_3_, 300 MHz) δ 0.86 (t, 7.2 Hz, 3H), 0.88 (t, 6.9 Hz, 3H), 1.25 (br, 32H), 1.54 (m, 4H), 1.89 (m, 4H), 2.32 (s, 3H), 2.52 (s, 3H), 2.98 (m, 1H), 3.09 (m, 1H), 3.25 (m, 1H), 3.63 (m, 1H), 4.05 (t, 6.3 Hz, 2H), 4.09 (t, 6.6 Hz, 2H), 6.52 (d, 7.8 Hz, 1H), 6.64 (s, 1H), 7.04 (m, 3H), 7.13 (d, 7.8 Hz, 2H), 7.36 (s, 1H) ppm; ^13^C NMR (CDCl_3_, 125 MHz) δ 14.1, 20.3, 20.8, 22.7, 26.2, 29.5 (m), 31.6, 31.9, 33.0, 35.0, 69.2, 69.4, 89.6, 89.7, 94.1, 94.9, 113.9, 116.0, 116.4, 1230, 125.7, 129.2, 129.3, 130.8, 132.2, 134.9, 136.4, 137.2, 139.3, 142.8, 153.4, 153.5 ppm. HRMS (MALDI) calcd. for C_100_H_136_O_4_ M^+^: 1401.0433, found 1401.0433. ^1^H and ^13^C NMR spectra are shown in Figures S3 and S4, respectively. GPC curves of *m*-**P1** and *m*-**D1** are shown in Figure S5.

## 3. Results and Discussions

**Scheme 1:**
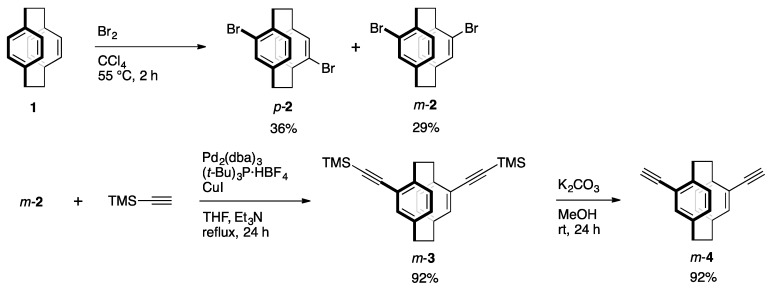
Synthesis of pseudo-*m*-diethynyl[2.2]paracyclophane *m*-**4**.

The synthetic route to the key monomer is shown in Scheme 1. Commercially available [2.2]paracyclophane (**1**) was reacted with Br_2_ in CCl_4_ at around 55 °C for 2 h to afford dibrominated [2.2]paracyclophanes. Pseudo-*p*-isomer *p*-**2** was precipitated during the reaction and could be obtained by filtration in 36% isolated yield. Pseudo-*m*-isomer *m-***2** existed mainly in the filtrate, and was isolated by recrystallization in 29% yield. Sonogashira-Hagihara coupling [21] of *m*-**2** with trimethylsilylacetylene proceeded smoothly using a Pd_2_(dba)_3_/(*t*-Bu)_3_P/CuI catalytic system to afford *m*-**3** in 92% isolated yield, and the successive removal of TMS groups by K_2_CO_3_/MeOH afforded *m*-**4** in 92% yield. Akita and coworkers reported the synthesis of *m*-**3** from *m*-**2** by the Sonogashira-Hagihara coupling using the PdCl_2_(PPh_3_)_2_/CuI catalytic system (33%) and the removal of TMS groups with Bu_4_NF to obtain *m*-**4** (79%) [18]; thus, the total yield of *m*-**4** from *m*-**2** was 26%. In addition, Hopf and coworkers independently synthesized *m*-**4** in total 55% isolated yield from *m*-**2** via pseudo-*m*-diformyl[2.2]paracyclophane [17]. Our present method improves the isolated yield of *m*-**4** from *m*-**2**, affording the total 85% yield. On the other hand, Lützen and coworkers reported the synthesis of optically active *m*-**4** from the corresponding enantiopure pseudo-*m*-diformyl[2.2]paracyclophane in high isolated yield, although chromatographic chiral resolution of the precursor was required [22].

**Scheme 2:**
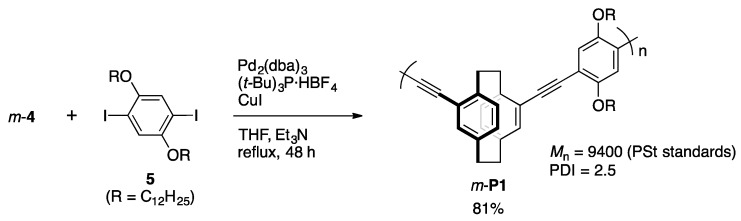
Synthesis of π-stacked polymer *m*-**P1**.

As shown in Scheme 2, the target polymer was synthesized via Sonogashira-Hagihara coupling using the same catalytic system as that of the synthesis of *m*-**3**. The reaction of *m*-**4** with diiodobenzene derivative **5** afforded the corresponding π-stacked polymer *m*-**P1** in 81% isolated yield. Once the polymer was precipitated, all of the polymer solid was not dissolved in solvents. The structure of the CHCl_3_-soluble part (84.2 wt/v %) of *m*-**P1** was confirmed by the ^1^H and ^13^C NMR spectra that were recorded in its CDCl_3_ solution. The number average molecular weight (*M*_n_) and polydispersity index (PDI) of the soluble part of *m*-**P1** in CHCl_3_ were estimated via gel permeation chromatography (GPC) to be 9400 and 2.5, respectively, using polystyrene (PSt) standards. A thin film was obtained from the CHCl_3_ solution by the casting or spin-coating method. The polymer showed thermal stability; 5 wt % weight loss was observed at 298 °C by TGA analysis (Figure S11, Supplementary Materials). As shown in Figure S12 (Supplementary Materials), in the DSC analysis, glass transition temperature (*T*_g_) and crystallization temperature (*T*_c_) were observed at around 60 and 170 °C by the first scan, respectively, whereas no peaks appeared in the second scan. Thus, the polymer showed sufficient thermal stability.

**Scheme 3:**
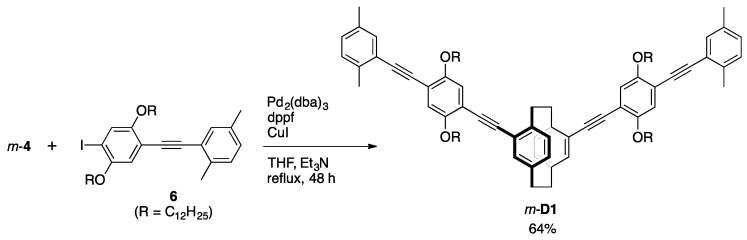
Synthesis of π-stacked dimer *m*-**D1**.

**Figure 2:**
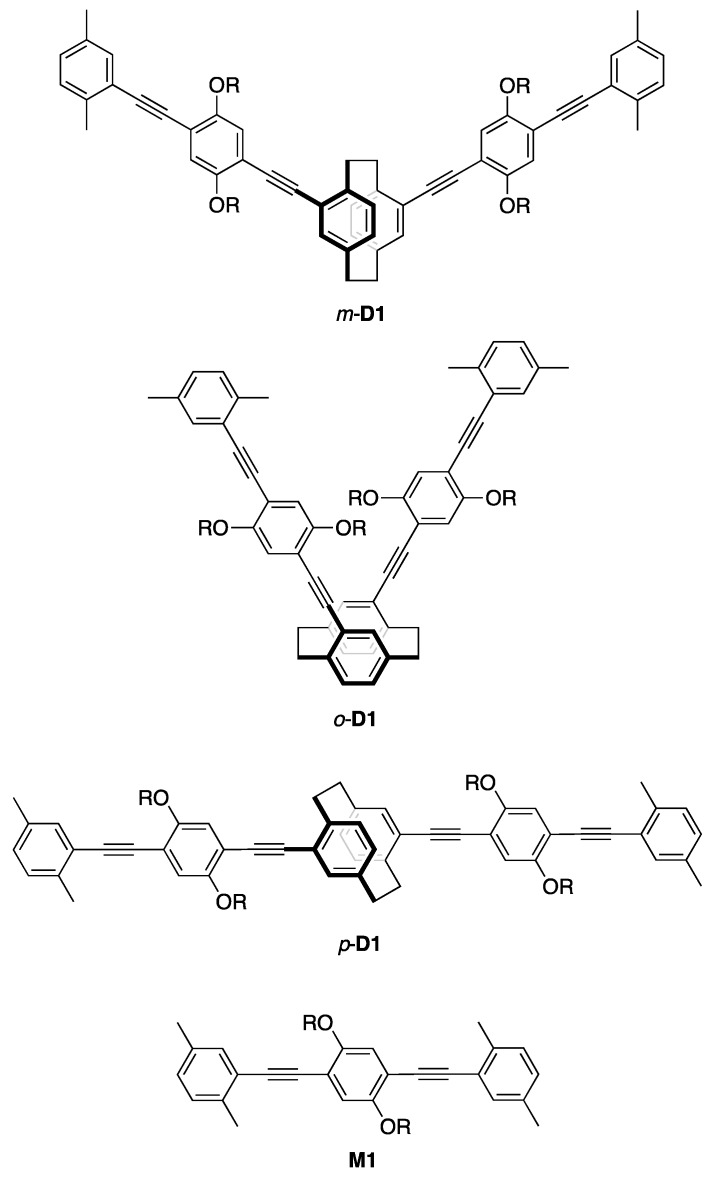
Structures of π-stacked dimers, *m*-, *o*-, *p*-**D1** and monomeric model compound **M1** (R = C_12_H_25_).

Scheme 3 shows the synthesis of π-stacked dimer *m*-**D1** as a model compound. The reaction of *m*-**4** with compound **6** using a Pd_2_(dba)_3_/dppf/CuI catalytic system afforded *m*-**D1** in 64% isolated yield, in which two PAE-type-π-electron systems are stacked at the terminal xylyl units. Previously, we prepared the stereoisomers of the π-stacked dimers, as shown in Figure 2. π-Stacked dimer *o*-**D1** comprises a pseudo-*o*-linked [2.2]paracyclophane skeleton, in which two PAE-type-π-electron systems are stacked and oriented at an angle of 60°. π-Stacked dimer *p*-**D1** incorporates the pseudo-*p*-linked [2.2]paracyclophane skeleton. Finally, we prepared **M1** as the monomeric PAE-type-model compound (Figure 2).

**Figure 3:**
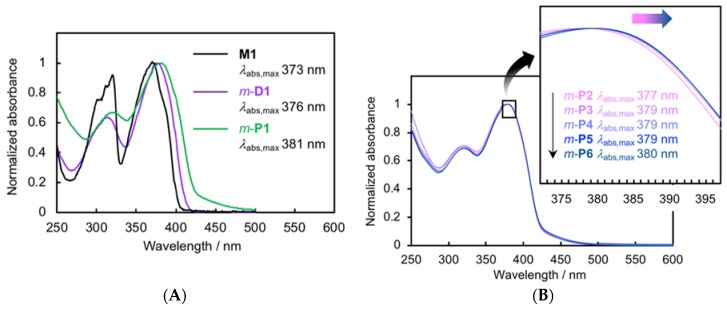
(**A**) UV-vis absorption spectra of **M1**, *m*-**D1**, and the soluble part of *m*-**P1** in CHCl_3_ (1.0 × 10^–^^5^ M for **M1** and *m*-**D1**, and 0.84 × 10^–5^ M for *m*-**P1**); (**B**) UV-vis absorption spectra of *m*-**P2**–**P6** in CHCl_3_.

Figure 3A shows the UV-vis absorption spectra of **M1**, *m*-**D1**, and the soluble part of *m*-**P1** in CHCl_3_ at room temperature. **M1** exhibited a typical π–π* absorption band with an absorption maximum (_abs,max_) at 373 nm. The absorption spectra of *m*-**D1** and *m*-**P1** are slightly red-shifted, with _abs,max_ values of 376 and 381 nm, respectively. The tailing band appeared in the spectrum of *m*-**P1** from 425 to 500 nm due to the intermolecular π–π interactions of the aggregated polymer chains. This tailing band was also observed in the *m*-**P1** casting film (Figure S8, Supplementary Materials). Polymer solution of *m*-**P1** in CHCl_3_ was separated by recyclable HPLC into five polymer solutions *m*-**P2**-**P6** with *M*_n_ values of 3800, 5000, 7000, 10,000, and 15,000, respectively. In their UV-vis absorption spectra of *m*-**P2**-**P6** shown in Figure 3B, minimal red-shift was observed as the *M*_n_ increased. For example, the _abs,max_ of *m*-**P2** (*M*_n_ of 3800) was 377 nm, whereas that of *m*-**P6** (*M*_n_ of 15000) was 380 nm. Thus, through-space conjugation was observed in the π-stacked polymers consisting of the pseudo-*meta*-linked [2.2]paracyclophanes. 

**Figure 4:**
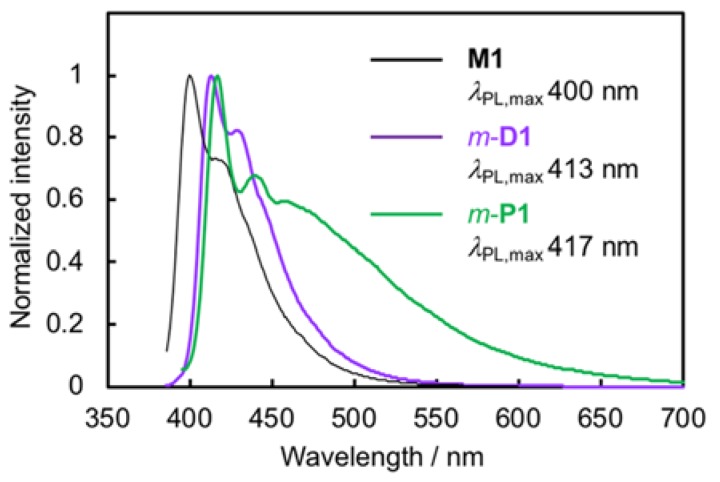
PL spectra of **M1**, *m*-**D1**, and the soluble part of *m*-**P1** in CHCl_3_ (1.0 × 10^–5^ M for **M1** and *m*-**D1**, and 0.84 × 10^–5^ M for *m*-**P1**), excited at each _abs,max_.

**Figure 5:**
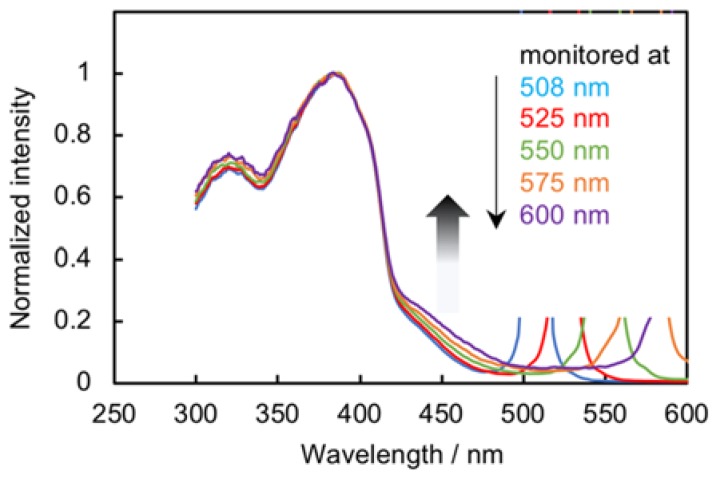
Excitation spectra of the soluble part of *m*-**P1** in CHCl_3_ (0.84 × 10^–5^ M) at room temperature, monitored at 508, 525, 550, 575, and 600 nm. Intensities were normalized at peak tops around 380 nm.

Photoluminescence (PL) spectra of **M1**, *m*-**D1**, and the soluble part of *m*-**P1** CHCl_3_ solutions were obtained by excitation at _abs,max_ at room temperature; the spectra are shown in Figure 4. **M1** emitted efficiently with a PL quantum efficiency (Φ_PL_) of 0.72; the PL spectrum exhibited a clear vibronic structure. The PL spectrum of *m*-**D1** was identical to that of **M1**, albeit red-shifted slightly with a Φ_PL_ of 0.73. The PL decay curve of *m*-**D1** was fitted with a single exponential relation and the lifetime value (τ) was 1.27 ns with χ^2^ = 1.00 (Figure S6, Supplementary Materials), which was almost same as that of **M1** (τ = 1.24 ns, χ^2^ = 1.00). Pseudo–*meta*–linked π-stacked-dimer *m*-**D1** emits from the chromophore state rather than from the phane state [23,24,25,26,27], in other words, simple PL from a PAE-type-monomeric unit is observed rather than an excimer-like emission from the two stacked π-electron systems. This behavior was identical to those of the pseudo–*para*– and pseudo–*ortho*–linked dimers (*p*-**D1** and *o*-**D1**) (vide infra). The PL spectrum of *m*-**P1** was similar to those of **M1** and *m*-**D1**; however, further PL was observed at longer-wavelength (~460 nm) in addition to the shorter-wavelength PL with vibronic structure, and the Φ_PL_ was estimated to be 0.11. In addition, a broad PL spectrum was obtained in the *m*-**P1** film (Figure S9, Supplementary Materials). The PL decay curve at 575 nm could be fitted with the double exponential relation with χ^2^ of 1.06, and the τ values were estimated to be 1.06 ns and 3.52 ns (Figure S7, Supplementary Materials). The longer lifetimes arose from the emission from the intermolecular π–π interactions of the aggregated polymer chains. The excitation spectra of *m*-**P1** were monitored at 508, 525, 550, 575, and 600 nm; namely, the broad PL band at the longer wavelength, as shown in Figure 5. As the monitored wavelength grew longer, the intensity of the tailing peak increased, which supports the tailing peak in the UV-vis absorption spectrum (Figure 3A). That of *m*-**P1** film exhibited the clear tailing peak at the longer wavelength (Figure S10, Supplementary Materials), which was monitored at around 540 nm. The broad longer wavelength peaks of *m*-**P1** (Figure 3A and Figure 4) are derived from the intermolecular π–π interactions of the aggregated polymer chains in the ground and excited states, respectively. 

**Figure 6:**
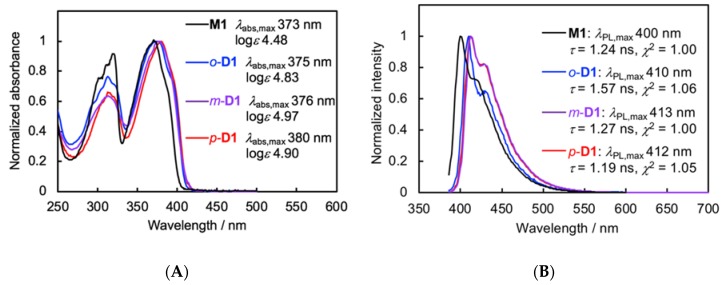
(**A**) UV-vis absorption spectra of **M1**, *o*-**D1**, *m*-**D1**, and *p*-**D1** in CHCl_3_ (1.0 × 10^–5^ M); (**B**) PL spectra of **M1**, *o*-**D1**, *m*-**D1**, and *p*-**D1** in CHCl_3_ (1.0 × 10^–5^ M), excited at each _abs,max_.

The optical properties of **M1** and the π-stacked dimers *o-*, *m-*, and *p-***D1** were compared, and the UV-vis absorption and PL spectra are shown in Figure 6A,B, respectively. The UV-vis absorption and PL spectra for the π-stacked dimers were red-shifted in comparison with those of **M1** due to the through-space conjugation. Essential differences in the optical properties among the dimers were not found: _abs,max_ was observed near 375 nm, and _PL,max_ appeared near 410 nm. These results indicate that the molecular orbital of the corresponding π-stacked unit is not influenced by the orientation of the π-stacked units despite the π-stacked structure in the ground as well as the excited state, although a small peak shift is observed in comparison with **M1** owing to the through-space conjugation. 

## 4. Conclusions

Pseudo–*meta*–diethynyl[2.2]paracyclophane, prepared by a modified synthetic method, was used as a monomer in the synthesis of a π-stacked PAE-type-polymer consisting of pseudo–*meta*–linked [2.2]paracyclophane. The absorption spectra of the π-stacked polymers after separation by recyclable HPLC as well as a π-stacked dimer were gradually red-shifted as the number of π-stacked units increased, indicating the through-space conjugation. The π-stacked polymer exhibited a broad absorption band derived from the intermolecular π–π interactions in addition to the π–π* transition band. PL from the intermolecular π–π interactions was also observed. No essential differences in optical properties among the π-stacked molecules comprising pseudo–*o*–, –*m*–, and –*p*–linked [2.2]paracyclophanes were observed. It was reported that pseudo-m-linked [2.2]paracyclophane is symmetry-allowed for electron transport [28]. The present polymer can be a promising candidate to function as a single molecular wire that transfers electron effectively. In addition, the pseudo–*m*–disubstituted [2.2]paracyclophane is optically active due to its planar chirality. The synthesis of optically active π-stacked polymers from enantiopure pseudo-*m*-disubstituted [2.2]paracyclophane is currently in progress. 

## Supplementary Materials

The following are available online at http://www.mdpi.com/2073-4360/10/10/1140/s1, Figure S1: ^1^H NMR spectrum of *m*-**P1**, Figure S2: ^13^C NMR spectrum of *m*-**P1**, Figure S3: ^1^H NMR spectrum of *m*-**D1**, Figure S4: ^13^C NMR spectrum of *m*-**D1**, Figure S5: GPC curves of *m*-**P1** and *m*-**D1**, Figure S6: PL decay curve of *m*-**D1**, Figure S7: PL decay curve of *m*-**P1**, Figure S8: UV-vis absorption spectrum of *m*-**P1** film, Figure S9: PL spectrum of *m*-**P1** film excited at 381 nm, Figure S10: Excitation spectrum of *m*-**P1** film monitored at 541 nm, Figure S11: TGA curve of *m*-**P1**, and Figure S12: DSC thermograms of *m*-**P1**.
