# Supplementary material for: π-Stacked Polymer Consisting of a Pseudo–meta–[2.2]Paracyclophane Skeleton
Source: Polymers (Basel). 2018 Oct 12;10(10):1140. doi: 10.3390/polym10101140 (PMC6403781; doi:10.3390/polym10101140)
Supplement: Supplementary file 1 [file polymers-10-01140-s001.zip › polymers-355405-SI.pdf]

Supplementary Materials: π-Stacked Polymer Consisting of a Pseudo–*meta*–[2.2]Paracyclophane Skeleton

Hazuki Maeda, Mayu Kameda, Takuji Hatakeyama and Yasuhiro Morisaki

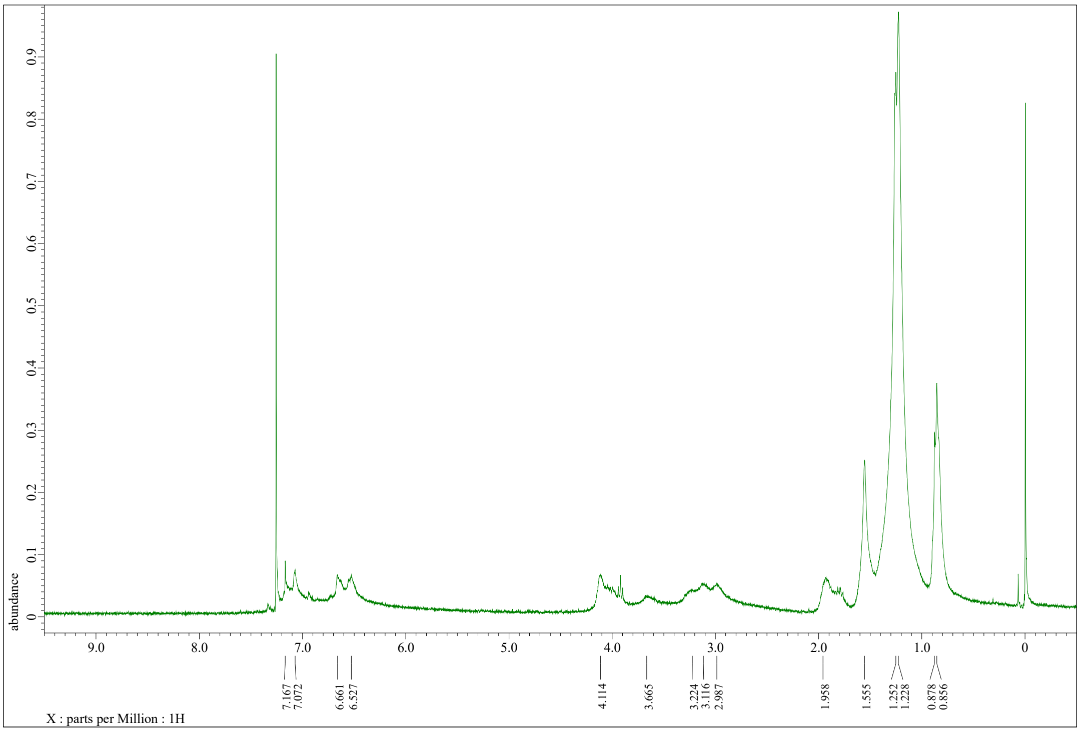

Figure S1. ^{1}H NMR spectrum of *m*-**P1**.

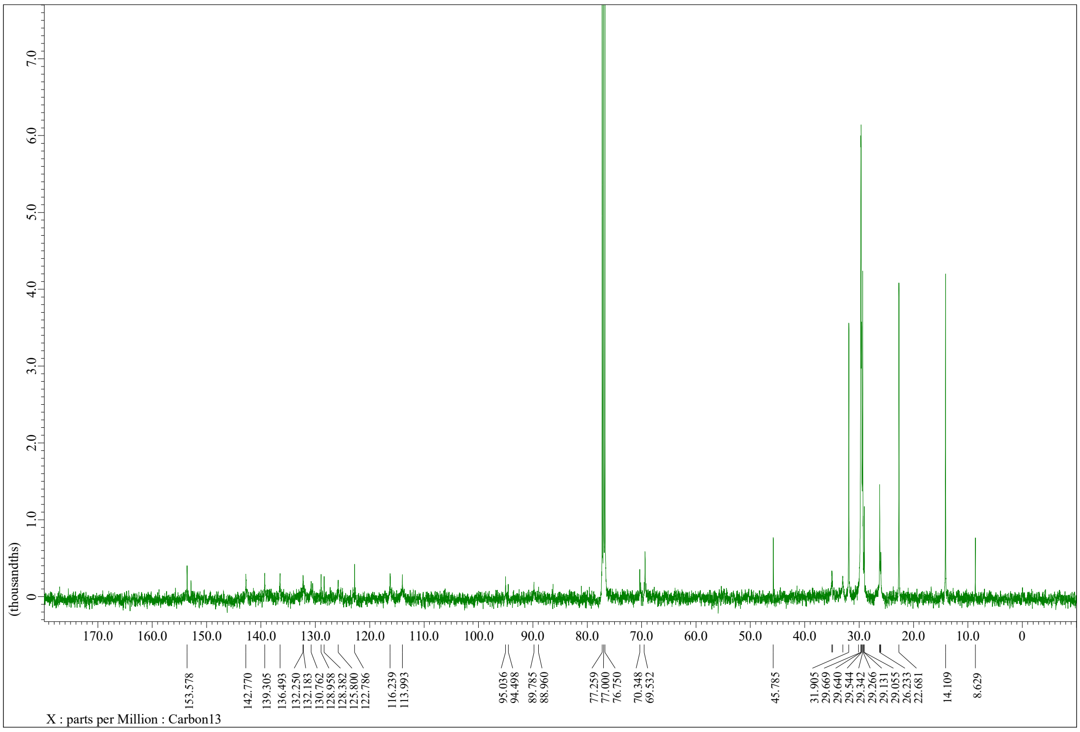

Figure S2. ^{13}C NMR spectrum of *m*-**P1**.

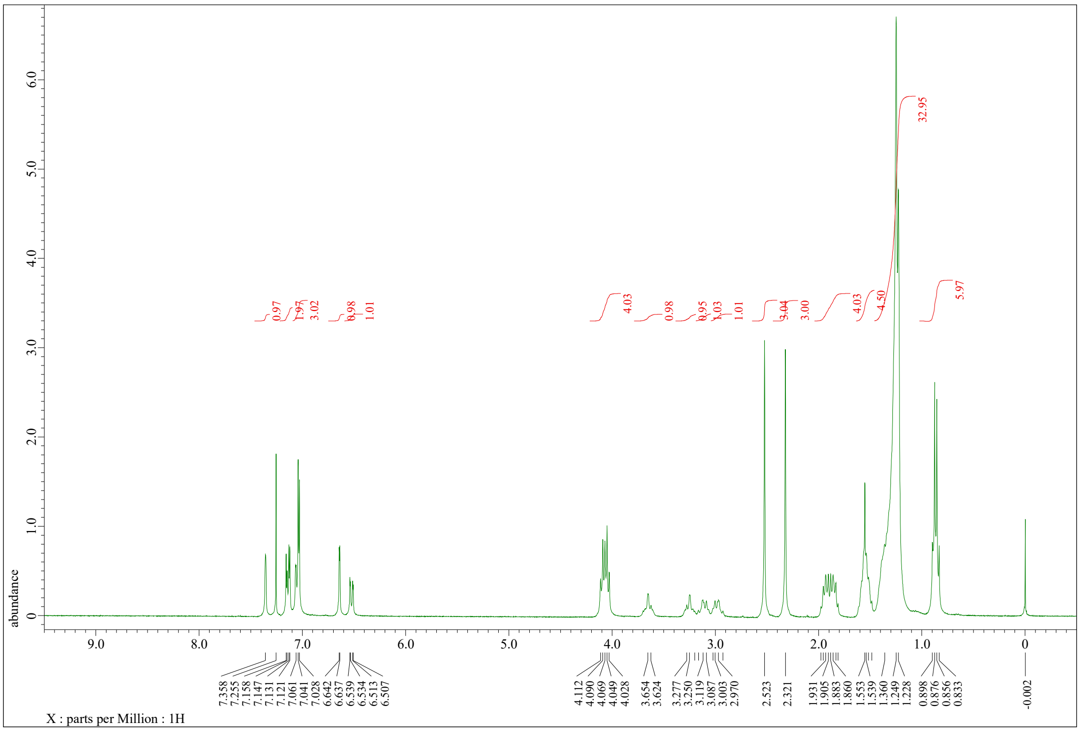

Figure S3. ^{1}H NMR spectrum of *m*-**D1**.

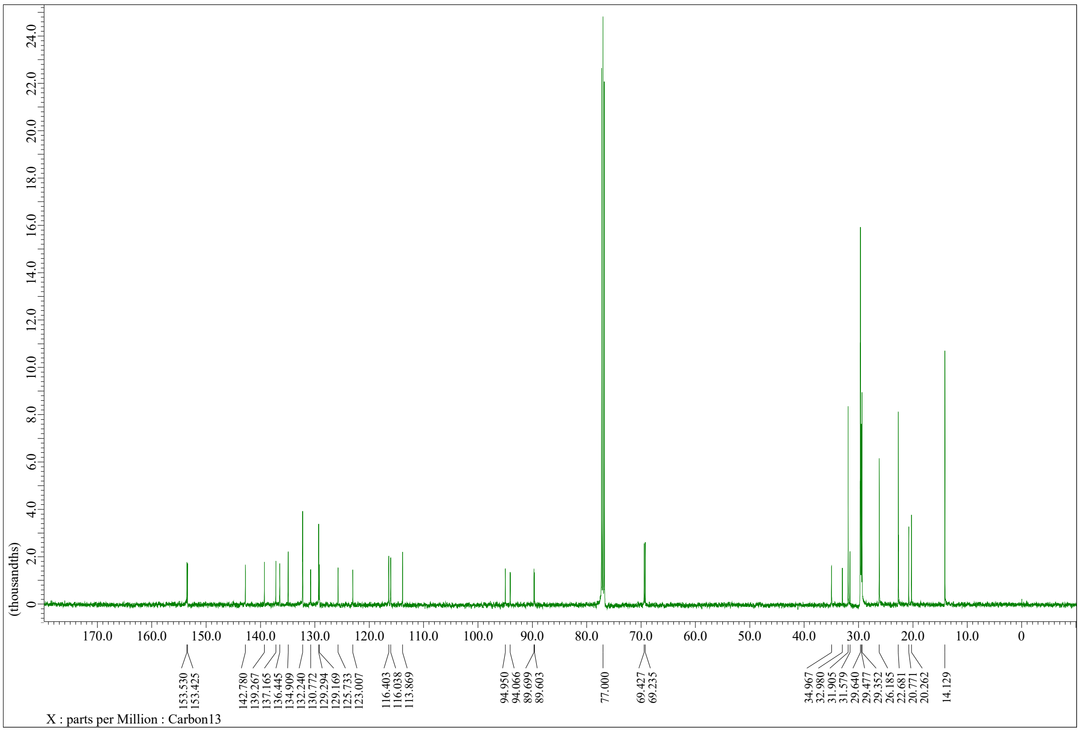

Figure S4. ^{13}C NMR spectrum of *m*-**D1**.

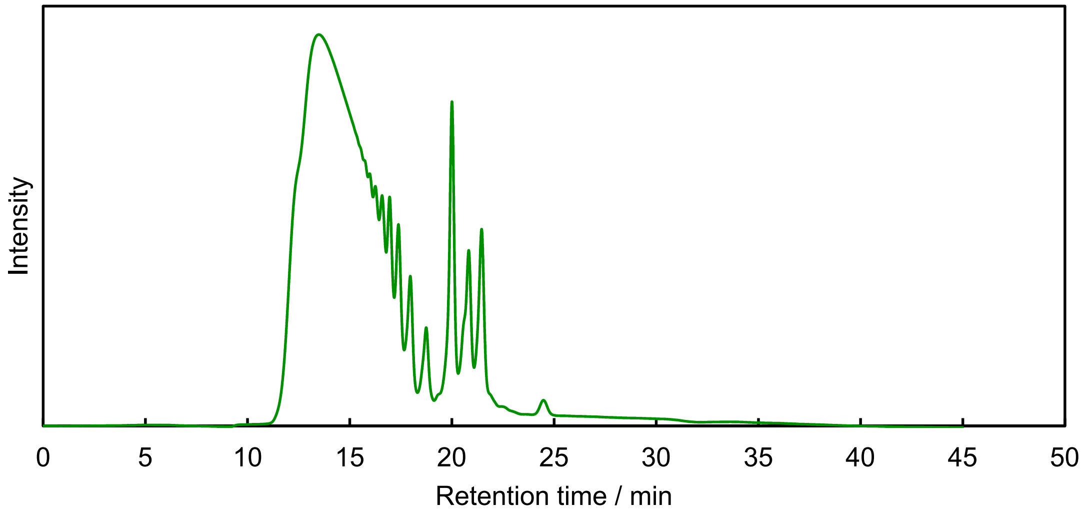

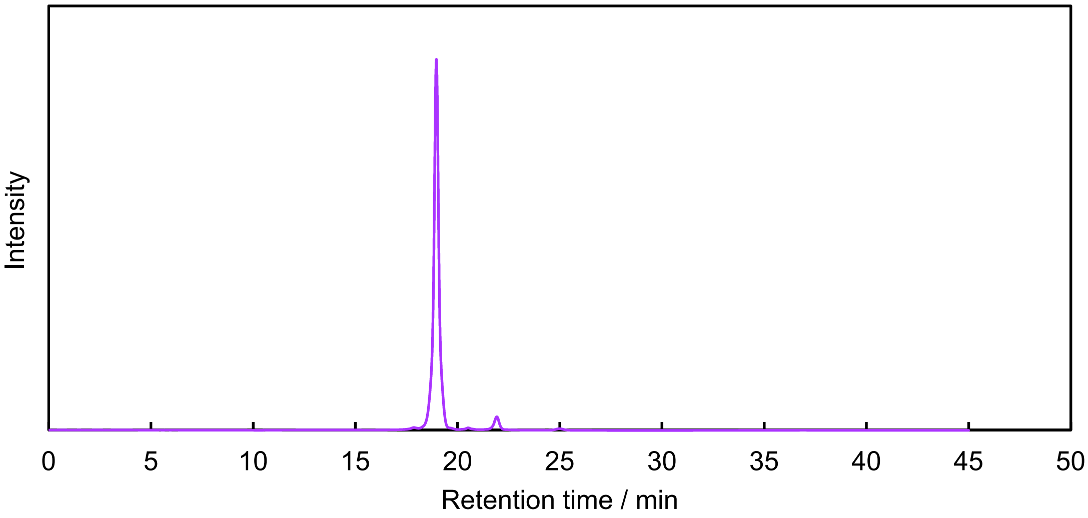

Figure S5. GPC curves of *m*-**P1** after the polymerization (upper) and *m*-**D1** (lower).

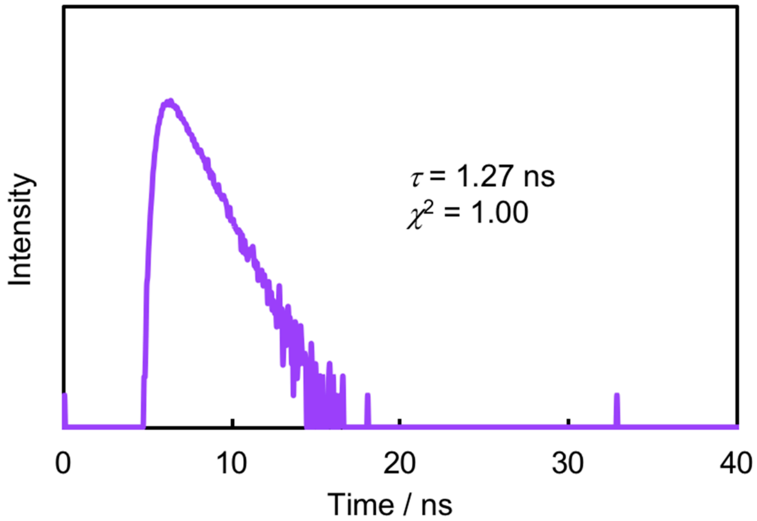

Figure S6. PL decay curve of *m*-**D1** at $\lambda_{PL,max}$ in $CHCl_3$ (1.0×10^{-5} M).

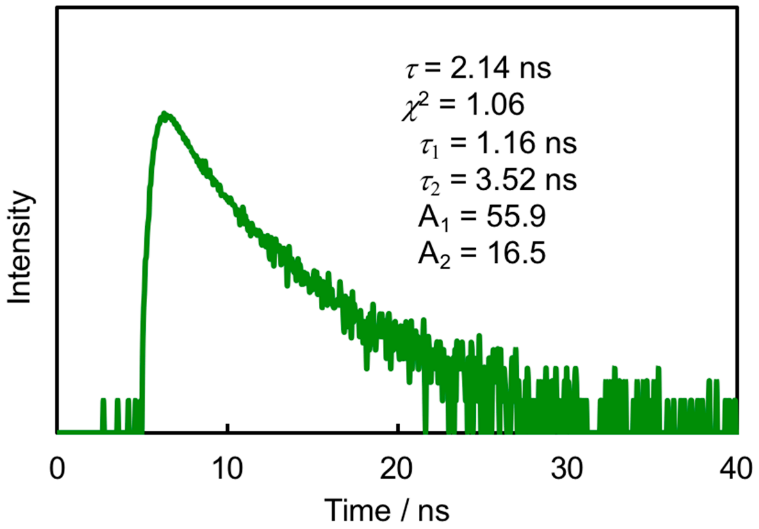

Figure S7. PL decay curve of *m*-**P1** at 575 nm in $CHCl_3$ (1.0×10^{-5} M).

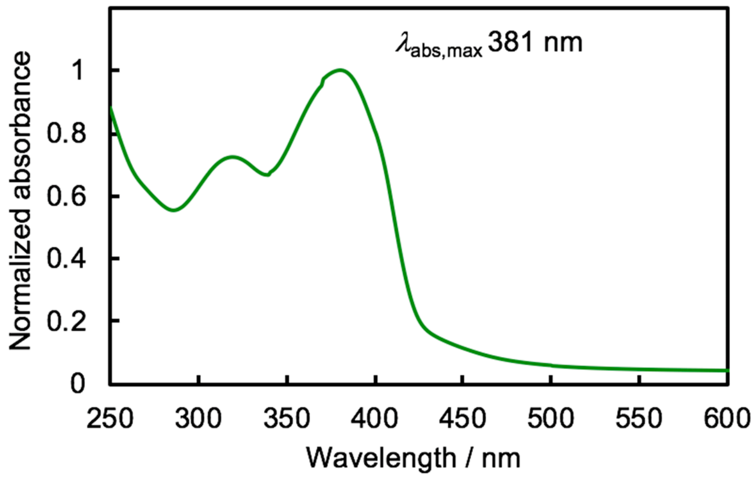

Figure S8. UV-vis absorption spectrum of *m*-**P1** film.

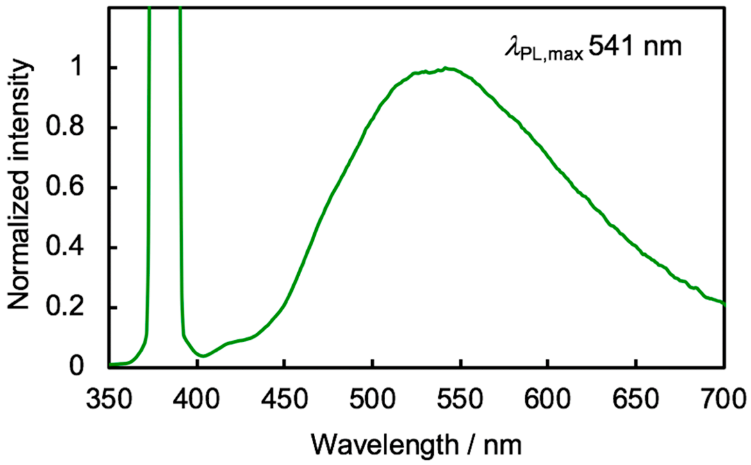

Figure S9. PL spectrum of *m*-**P1** film excited at 381 nm.

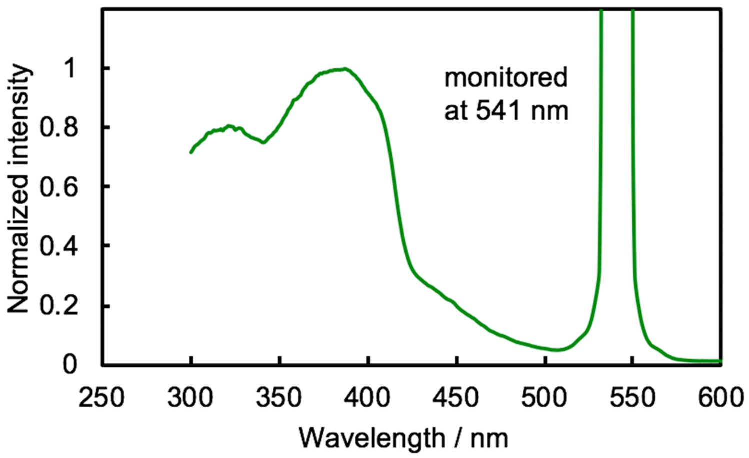

Figure S10. Excitation spectrum of *m*-**P1** film monitored at 541 nm.

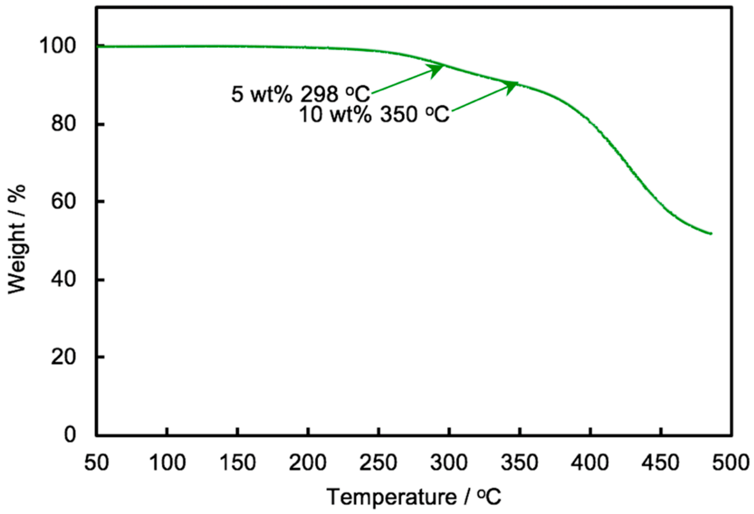

Figure S11. TGA thermogram of *m*-**P1** at heating rate of 10 °C/min under N_2.

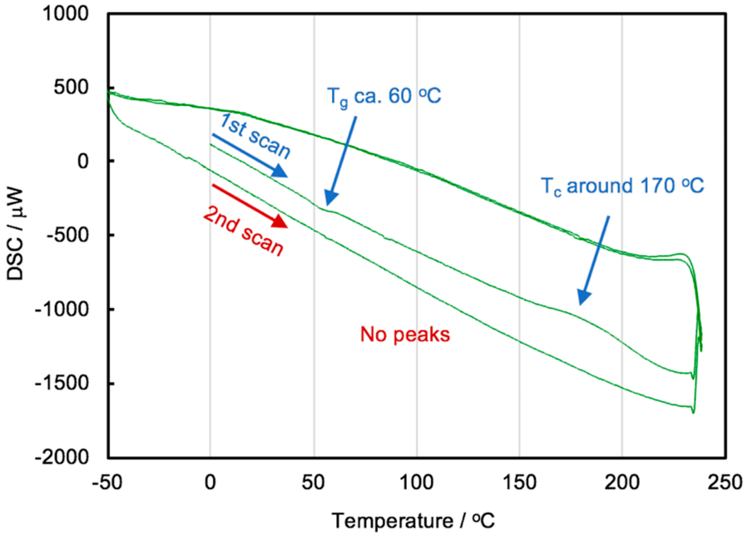

Figure S12. DSC thermograms of *m*-**P1** at heating and cooling rate of 10 °C/min under N_2.
